# Numerical Modeling Reveals That Resistant Western Corn Rootworm Are Stronger Fliers than Their Susceptible Conspecifics

**DOI:** 10.3390/insects15110834

**Published:** 2024-10-24

**Authors:** Katarina M. Mikac, Darija Lemic, Ivana Pajač Živković, Jose H. Dominguez Davila

**Affiliations:** 1 Environmental Futures, School of Earth, Atmospheric and Life Sciences, University of Wollongong Australia, Northfields Avenue, Wollongong, NSW 2522, Australia; kmikac@uow.edu.au; 2 Department for Agricultural Zoology, Faculty of Agriculture, University of Zagreb, 25 Svetošimunska, 10000 Zagreb, Croatia; ipajac@agr.hr; 3 South Coast Structural Engineers, P.O. Box U9, Wollongong, NSW 2500, Australia; jose_d@scsengineers.com.au

**Keywords:** finite element method (FEM), Bt-Corn resistant, rotation resistant, wing shape, aspect ratio

## Abstract

The hindwing geometry, aspect ratio, and numerical modeling of susceptible, Bt-Corn- and rotation-resistant western corn rootworm (WCR) wings was investigated. All variants had similar hindwing geometries and aspect ratio (AR: 6–7). These AR values correspond to wings suited to lower altitude flights of a shorter distance. These AR values are characteristic of wings that can carry heavier loads and are capable of precision flying. Numerical modeling using the finite element method (FEM) showed that the Bt-Corn-resistant and rotation-resistant WCR hindwings could potentially resist higher wind speeds with minimal deformations compared to conspecific susceptible WCR. Understanding the physiology and dispersal of resistant WCR enables a better understanding of how these variants spread their alleles across large scale agricultural landscapes. This may have important implications for integrated resistant management strategies for WCR.

## Simple Summary

The study investigated the hindwing geometry, aspect ratio, and flight capabilities of three western corn rootworm (WCR) types: susceptible, Bt-Corn resistant, and rotation resistant. All had similar wing structures suited for low-altitude, short-distance flights, allowing them to carry heavier loads and fly with precision. Numerical modeling showed that resistant WCR, especially Bt-Corn-resistant beetles, had wings that could better withstand higher wind speeds with minimal deformation compared to susceptible WCR. This suggests that resistant WCR are more likely to spread their alleles over larger agricultural areas under a range of prevailing wind conditions, possibly impacting resistance management strategies.

## 1. Introduction

The western corn rootworm (WCR), *Diabrotica virgifera virgifera* LeConte (Coleoptera: Chrysomelidae) is one of the major insect pests of corn in the USA and Europe. WCR has developed resistance to chemical, soybean-maize crop-rotation, and transgenic control practices such that its control is ever changing to combat the associated evolutionary changes the insect undergoes in parallel [1,2,3,4,5,6,7,8,9,10]. WCR invasion and its damage to corn production areas in the USA and Europe have been the subject of many studies since it first detection in corn in 1909 in the central Great Plains region of the USA [11].

Ongoing monitoring of WCR, including a greater emphasis on understanding its movement biology and whether resistant strains differ in their dispersal behavior and capacity, is needed [12]. Fundamental to the success of control programs are current data on the biology of a species; this is especially true for the WCR as there are now several resistant variants in the US that are known to differ in morphological traits critical to dispersal success [13,14,15,16,17,18,19,20]. Over the past decade there have been a series of studies that have demonstrated repeatedly changes in WCR wing shape and size as a function of their resistance to control [16,17,21]. While these studies show clear patterns associated with control practices, what these studies do not investigate is how WCR are phenotypically changing to accommodate their managed habitats (i.e., corn fields) while meeting their inherent dispersal requirements and long-term survival.

It is well documented that WCR dispersal is tied to their long-term survival and overall strategy for invasion success. It has been shown that this significant beetle pest can undertake long-distance flight of 24 km (one flight) or a total of 39.6 km across a 24 h period [22]. The beetle has been shown during flight mill studies to fly anywhere between 150 min [22] to 240 min [23]. This flight capacity coupled with the assistance of local weather conditions, especially storms, has been the likely reason that WCR have been such successful invaders over large geographic areas in the USA and Europe. Isard et al. [24] showed that rotation-resistant WCR flight mostly occurred in the morning from 06:45–11:00 a.m. and afternoon and evening from 05:00–8:30 pm by newly mated females. In their previous work, Isard et al. [25] suggested that flight in WCR from 07:00–09:00 a.m. was greatest to coincide with weather conditions in the lower atmosphere which would be most unstable at that time, thus possibly providing rotation-resistant WCR added flight distance advantage by passive dispersal (e.g., susceptible WCR long-distance dispersal aided by storms [26]). Earlier studies showed that WCR vertical flight behavior increased during 07:00–09:00 a.m. and 01:00–03:00 p.m. [27], again likely to coincide with prevailing weather conditions that can assist them in long-distance dispersal [26]. Importantly Isard et al. [24] showed that of the 85% of rotation-resistant WCR caught undertaking high altitude flight, 99% were mated, corn fed females that evidently capable of dispersing their resistant alleles across large agricultural landscape scales.

Clearly, WCR have the capacity for long-distance dispersal, though no current data are available on whether resistant variants, e.g., Bt-Corn-resistant WCR, are also capable of long-distance dispersal. Further to this is a lack of understanding on WCR variants and their flight abilities during a range a wind conditions. While there are studies conducted on their use of daily summer weather patterns, storms in particular, for long-distance dispersal [26,27], Isard et al. [24] suggested that rotation-resistant WCR only flew when wind speeds were 0.2 and 1.1 m/s and not above 2.1 m/s. WCR flight at higher wind speeds and adverse weather conditions remains unknown, even though it is an important contributor to their long-distance dispersal strategy [26]. Alternative and proven methods to numerically model the flight of WCR variants across a range of wind conditions should be explored.

**Figure 1:**
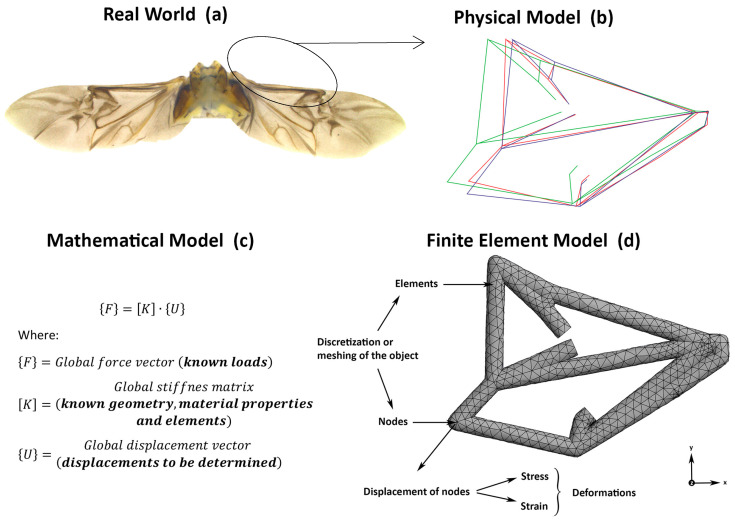
Workflow diagram of a typical numerical modeling analysis using the finite element method (FEM): (**a**) starting with a real world problem or object (Bt-Corn-resistant western corn rootworm wingspan); (**b**) that is transformed into a physical model (venation shape wireframe of resistant and susceptible western corn rootworm); (**c**) from which mathematical characterization of the object (here a wing) subjected to forces and depending on its material properties, and shape elements is obtained; (**d**) and finally through a finite element model, displacement is obtained and the stresses and strains (collectively referred to as: ‘deformations’) determined. Load was applied on the z-axis.

Numerical modeling via the finite element method (FEM) has been used to examine flight potential and behavior in insects primarily to understand how wings as structures adjust to different loads (wind) and load combinations [28,29,30,31,32,33]. The basic concept behind the FEM is to replace any complex shape (i.e., insect wings) with the union (or summation) of many simple shapes (like triangles) that are combined to accurately model the original shape (i.e., wing or venation shape). These now smaller and simpler shapes are called ‘finite elements’ because each one occupies a small but finite sub-domain of the original part. From here, it is possible to understand how wings as structures perform under any combination of loading scenarios (Figure 1). One of the better-known examples of numerical analysis of insect flight is that of Combes and Daniel [29,30], who used the FEM to investigate flexural stiffness distribution of hawk moth, *Manduca sexta* wings in response to aerodynamic forces applied dorsally and ventrally. These authors found that the greatest level of deformation occurred on the outer distal edges of wings where aerodynamic forces can be accommodated by the wing structure.

Mostly, numerical modeling of insect flight has application in mechanical and materials engineering, though Wootton et al. [31] used the FEM extensively to investigate the evolution of flight in insects and the resulting physiological/mechanical changes and implications for insect dispersal. The FEM has been used by many authors who have examined the importance of aerodynamic forces applied to insect wings to understand the stress–strain relationship of the wing and how this affects insect flight [28,29,30,31,32,33,34,35,36]. From this body of research, it is evident that insect wing veins provide effective support to the wing membrane and are critical in mitigating buckling stress or failure during flight [29,32,33,37,38,39,40,41]. Further, variation in wing venation and shape can be used for quantitative characterization of insect flight potential and behavior and thus can provide detailed insight on an insect’s dispersal ability [16,18,21,36,42], which can be difficult to investigate under field conditions.

Here, we investigate WCR conspecific flight capacity of Bt-Corn- and rotation-resistant and susceptible beetles. We do this by analyzing wing geometry, in addition to numerical modeling of hindwing shape differences based on resistance type. The use of these methods will enable a greater understanding of WCR physiology and dispersal capabilities including the potential for spread of resistant alleles across large geographic scales.

## 2. Materials and Methods

### 2.1. Sample Collection

WCR resistant to Bt-Corn (*n* = 35), soybean-maize rotation (*n* = 120) and non-rotation resistant (susceptible) variants (*n* = 70) were collected from across multiple sites in Iowa, Indiana and Illinois, USA, with the specifics of their collection methods and locations previously reported [18].

### 2.2. Wing Geometry and Aspect Ratio (AR)

The left and right wings and pronotum of individual WCR from populations stated above were dissected and slide mounted using standard slide preparation procedures [43]. ImageJ v1.46r was used to measure wing length (mm), width (mm) and area (mm) and pronotum width (mm) from the WCR populations stated above. Aspect ratio was calculated as wingspan^2^/wing area (dorsal surface of wing only) [44].

### 2.3. Numerical Modeling: Finite Element Method (FEM)

The FEM is a computer-based numerical method for solving three-dimensional problems with complicated geometries, loadings, and material properties (Figure 1). There are scenarios in which analytical solutions cannot be obtained to identify and predict the performance and behavior of complex objects such as wings, therefore the FEM can be used. Using the FEM, stresses (wind loads) and deformations (wing deformations) can be determined using a predetermined model (Figure 1). The FEM was used to examine flight capacity in WCR. The deformation of WCR forewings was based on a range of wind speeds experienced in the field (outlined below), which were numerically modeled from the three treatments described above (rotation resistant, Bt-Corn resistant and susceptible). Three simplified finite element models of the WCR forewings (based on treatments) were tested.

Prior to the FEM, a choice between the use of rigid and flexible wing models was made. The choice between rigid and flexible wings analysis depends on the specific aerodynamic needs of the study model, with rigid wings offering stability and predictable performance [45]. Here we used rigid models particularly in the context of stress and deformation, because of structural integrity and predictability in stress distribution under aerodynamic loads [45].

ANSYS workbench software v19 (ANSYS^®^ Academic Research Mechanical, Release 19.0, ANSYS Inc., Canonsburg, Pennsylvania, USA) was used to create a simplified 3D finite element model of wing shape (based on wing venation patterns) and the symmetric vein shape bending deformation or flexural stiffness (relationship between stress and strain) of each WCR variant was tested (Figure 1). The 2D vein shape model was generated from 14 landmarks identified on WCR veins using TPSDIG v2016 [46]. To generate wireframes after Procrustes analysis, MorphoJ v1.06d was used [47]. Then, a computer-generated image of the wireframe average shape of WCR was modeled using computer-aided design (CAD). The 2D wireframe CAD models were introduced in ANSYS workbench to generate a 3D skeleton model for static structural analysis (Figure 1). The three-dimensional skeleton model (wing structures) were loaded using minimal to maximum wind speeds and converted to pressure magnitude. Wind speed was converted to wind pressure using the dynamic pressure formula:

$$P=\frac{1}{2}\rho v^{2}$$

where P is the wind pressure, ρ is the air density, and v is the wind speed. This wind pressure (force per unit area) was then applied over the surface of the 3D wing along the *z*-axis in the FEM simulations to model the aerodynamic load [30,48]. A maximum wind speed of 4.2 m s^−1^ was used, as this is double the wind speed that others had found that rotation-resistant WCR no longer flew in the field [24]. To analyze the bending asymmetry of the wing under such wind conditions, the boundary condition or fixed support (no rotation) was located at the base (i.e., humeral and axillary plates) of the wing.

The material properties of the wing vein models were based on previous studies [30,35,36,48]. The wing vein material was characterized as an isotropic elastic with an element density (*ρ*) of 1200 kg m^−3^ and element thickness (t) of 45 µm. A Young’s modulus (E) of 150 MPa was used to characterize the stiffness of the vein material. Finally, a Poisson’s ratio (v) of 0.3 was used. The FEM flexural stiffness (maximum deformation) was evaluated using linear and non-linear static solutions to compare the smallest and largest deformations (applying wind speed loads from 10 km/h to 100 km/h) of the three WCR wing venation patterns. Deformations were controlled by the venation shape, the material stiffness (Poisson’s ratio and Young’s modulus), physical properties (density), and diameter of the veins [30,48].

To characterize the bending behavior of the three WCR variants’ venation shapes, an ANSYS mesh was generated using 6454 cubic elements with 11,135 nodes to discretize and resemble the wing geometry. Mesh verification, an essential step in ensuring simulation accuracy, includes not only convergence assessment but also additional measures to ensure mesh quality. While convergence, where further mesh refinement no longer significantly alters the results, was achieved through iterative mesh refinement in ANSYS, additional steps were taken to verify mesh quality. These included checking the element aspect ratio, ensuring the elements were well-shaped (not distorted), and confirming that the mesh was sufficiently fine in critical areas, such as near the connections between the veins, where deformations are more complex. Additionally, the mesh verification process involved comparing the results with known benchmarks or analytical solutions where applicable, ensuring that the simulation’s accuracy extended beyond simple numerical convergence. This comprehensive approach ensured that both the geometric representation of the wing and the resulting deformations were reliable, offering confidence that further mesh refinement would not significantly change the outcome. By addressing both convergence and mesh quality, the FEM simulations produced accurate output, crucial for analyzing the aerodynamic and structural behavior of the modeled wings (Figure 1d).

## 3. Results

### 3.1. Wing Geometry: Aspect Ratio (AR)

Aspect ratio was consistent among the three variants investigated and had a mean range from AR = 6 for rotation-resistant and susceptible beetles, to AR = 7 for Bt-Corn-resistant WCR. 

### 3.2. Finite Element Method (FEM)

**Figure 2:**
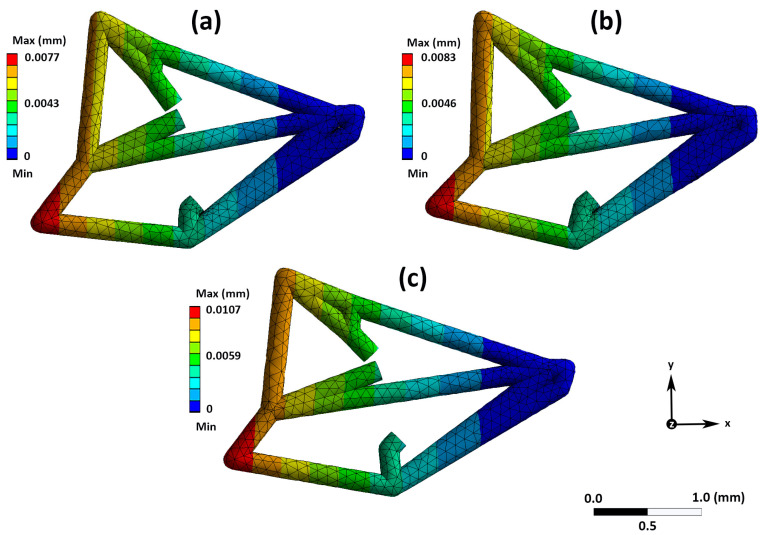
Wing deformation (mm) modeled against a maximum wind speed of 4.2 m/s using the finite element method for all western corn rootworm (WCR) along the *z*-axis: (**a**) Bt-Corn-resistant WCR wings; (**b**) rotation-resistant WCR wings; (**c**) susceptible WCR wings. Load was applied on the z-axis.

Static bending tests (or deformation), which occurred by fixing wings at their base (i.e., deep blue colored part of vein structure; Figure 2), provided a gradient of deformation from the base of the vein to the distal tip of the beetle vein structure (red colored part of vein: Figure 2). The maximum deformation modeled for all beetle wings along the *z*-axis ranged from 0 (dark blue color: Figure 2) to 0.0107 mm (red color: Figure 2) under a maximum wind speed (pressure force) of 4.2 m/s. Specifically, Bt-Corn-resistant WCR wings had a structural vein deformation of 0.0077 mm (29%) under maximum wind conditions (Figure 2a). Similarly, for rotation-resistant beetles, structural vein deformation was 0.0083 mm (31%) (Figure 2b). Finally, the largest deformation (0.0107 mm or 40%) was found for susceptible WCR variant wings under the same maximum wind conditions stated above (Figure 2c).

These structural deformations were further visualized via a representative numerical model of a loaded wing under maximum wind speed conditions (Supplementary Materials: Video S1). In the model, deformation is seen across the vein structure through a color change progression from blue (zero deformation) to red (maximum deformation) (Supplementary Materials: Video S1).

**Figure 3:**
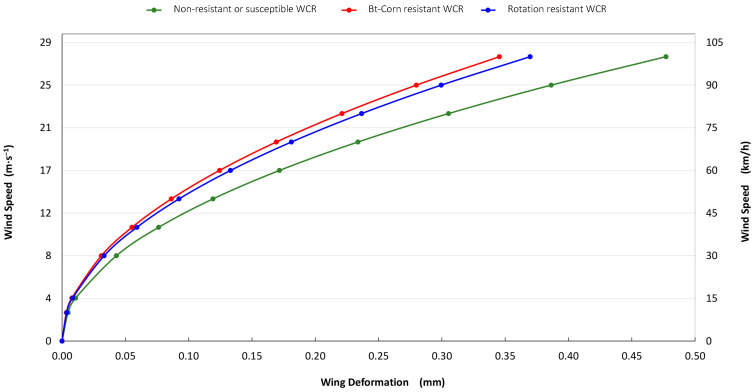
Finite element method modeled wing deformation (mm) versus wind speed (m/s) of the three western corn rootworm variants investigated.

Finally, wing deformation versus a range of wind speeds were modeled for the three variant beetle types (Figure 3). Incorporated in the model were the minimum and maximum wind speeds that WCR are exposed to under field conditions. The Bt-Corn- and rotation-resistant beetles had the least wing structural deformation across a range of extreme wind speeds, compared to susceptible beetles (Figure 3). Bt-Corn-resistant beetles had the least wing structural deformation across the range of wind speeds modeled for all beetle types, and their wing structures were robust even at speeds where WCR are known from field experiments to no longer fly.

## 4. Discussion

Here, we found that all three WCR types investigated had similar mean AR values (6–7). Wing geometry principles state that a longer and thinner wing shape will have a higher AR (>10) and such a wing will have capacity for greater lift during take-off, long endurance or sustained flight, stability (though not maneuverability), and less drag during flight (i.e., less energy loss). By contrast, a shorter and more rounded wing shape will have a lower AR value (<10), and such a wing will have capacity for lower altitude and shorter flights, more maneuverability (flight precision), more stability, and better capacity for carrying heavier payloads (e.g., female WCR with eggs).

The AR values <10 found here suggest that all three WCR variant types have wings suited to shorter distance and more maneuverable flights, with the ability to carry heavier loads if needed. Studies specifically examining long-distance flight in insects have shown marked differences in conspecific wing shape depending on whether populations are short distance, migratory or non- migratory dispersers, i.e., milkweed bugs [49] and monarch butterflies [44,50]. Mikac et al. [17,18,21] have shown significant differences in wing size and shape for susceptible compared to resistant WCR variants and demonstrated that the wings of resistant individuals are shorter and more rounded than the longer and thinner wings of their susceptible conspecifics; these findings correspond with our findings reported here. A shorter and more rounded wing shape is likely able to assist both Bt-Corn- and rotation-resistant WCR to undertake inter-field strategic flights, with higher payloads (gravid females), across a greater range of wind speeds.

Through the FEM modeling, resistant WCR had the lowest wing structural deformations even at wind speeds that far exceed those at which they were observed to no longer fly [24]. Through numerical modeling, we demonstrated that the wings (venation shape) of the Bt-Corn- and rotation-resistant WCR showed more resistance with less deformation (29 and 31%, respectively) under even extreme wind conditions. Susceptible WCR showed the largest deformation under the same wind conditions (40%), possibly leaving them more disadvantaged for long-distance flight across a range of wind speeds [24].

The collective importance of our data on wing physiology and structural integrity is that resistant WCR have a wing shape that enables them to be more effective dispersers since they can carry heavier loads and undertake flights with greater precision and maneuverability, allowing them to have more control over their flight and thus dispersal. Our most significant finding is that the wings of resistant WCR genotypes, true for both Bt-Corn- and rotation-resistant beetles, are potentially structurally stronger than susceptible conspecifics and able to withstand even extreme wind speeds.

The structural engineering analysis of insect flight we undertook here, while focusing on key parameters such as stress and deformations, provides a simplified but valuable framework for understanding one aspect of the complex interactions involved in insect flight. Although it is true that flight encompasses multiple factors, such as wind, turbulence, aerodynamics, and energy consumption, structural integrity of the wing is a critical element. It determines the insect’s capacity to withstand forces during flight, making it a key element for any further exploration of flight dynamics in a species. The conclusions we draw about WCR flight are derived from isolating the structural effects, which, while representing only one aspect of the broader dynamics of flight, are crucial for understanding overall flight performance in the species. Nevertheless, our focus on wind pressure and WCR wing structural integrity provides a benchmark perspective, essential for future work that can build on this to examine and model complex interplay of multiple additional parameters and forces that potentially influence flight in insects [30].

These novel findings on the wing physiology and flight behavior of resistant WCR have important implications for integrated resistance management given that these variants may have increased flight capabilities, and thus can effectively spread resistant alleles across large geographic potentially irrespective of the prevailing wind conditions. Future work should focus on experimental flight of Bt-Corn-resistant and rotation-resistant WCR to better understand contemporary flight and dispersal behavior in resistant WCR (e.g., flight mill or wind tunnel). An overall deeper understanding of wing physiology, flight, and dispersal behavior in WCR, which are ever evolving to control practices, will enable the implementation of current data-driven integrated resistance management strategies for the species.

## 5. Conclusions

The three WCR types investigated in this study had similar wing geometries all suited for flights where greater payloads can be accommodated and where maneuverability is optimal. The FEM analysis showed that resistant (especially Bt-Corn-resistant beetles) WCR wing shapes and venation patterns may resist higher wind speeds with minimal deformations compared to non-resistant (susceptible) WCR.

## Data Availability

The data supporting the findings of this study are available upon reasonable request from the corresponding author.

## Supplementary Materials

The following supporting information can be downloaded at: https://www.mdpi.com/article/10.3390/insects15110834/s1, Video S1: A representative numerical model of a loaded western corn rootworm wing under maximum wind speed (4.2 m/s) conditions.
